# Supplementary material for: Identification of Freezing-Responsive microRNAs and Their Targets in Chinese Jujube by Small RNA and Degradome Sequencing
Source: Int J Mol Sci. 2026 Sep 4;27(17):7912. doi: 10.3390/ijms27177912 (PMC13566890; doi:10.3390/ijms27177912)
Supplement: Supplementary file 1 [file ijms-27-07912-s001.zip › Supplementary Material S2/novel_miR_67.pdf]

Provisional ID	:	novel_miR_67
Score total	:	0.5
Score for star read(s)	:	-1.3
Score for read counts	:	0
Score for mfe	:	0.2
Score for randfold	:	1.6
Score for cons. seed	:	
Total read count	:	245
Mature read count	:	245
Loop read count	:	0
Star read count	:	0

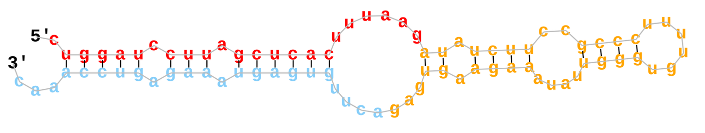

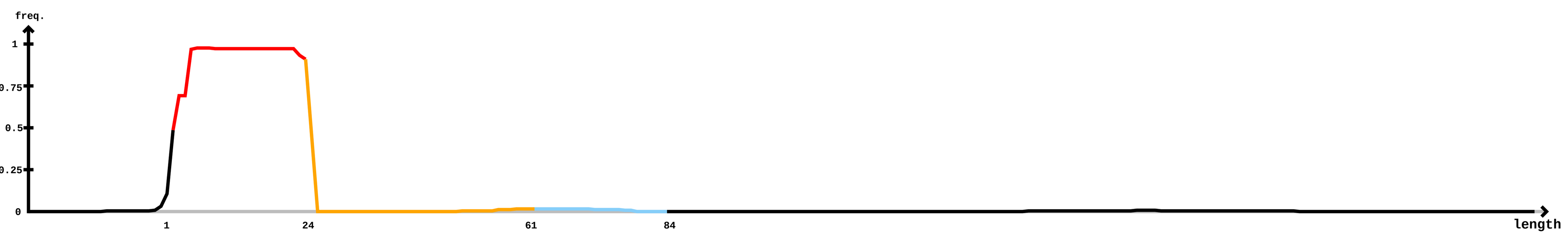

Mature Star

```
5'- auuuaaacucuuauuuaauuuuucuggauccuuagcucacuuuaagauaucuuccgcccuuuuguggguuauaaagaagugagacuugugaguaaagaguccaaacacguaaguugauuuuggaucagaauaaacaauuuguuuugaccugauuguaagauucuucauucuauuauucugauucauucauaaugcagcgagguggugauccgcucauucuuuguuucguggauuuugcaaauccagccugu   -3'   exp
    ..........(((((.......(((((.(((.((((((......((.((((..((((......))))....)))).))......)))))).))).)))))......)))))(((...((((((((((((((((((.((....))..)))))).(((........))).))))))))))))).))).((((.((((.(((....)))))))))))..........(((((((...)))))))......   reads   mm   sample
    ..........auuuaauuuuucuggauc.......   1    0   LT-1
    ..................uuucuggauccuuagcucacuuuaag.......   1    0   LT-1
    ...................uucuggauccuuagcucacuuuaag.......   1    0   LT-1
    ...................uucuggauccuuagcucacuuuaag.......   4    0   LT-2
    ...................uucuggauccuuagcucacuuuaag.......   1    0   JCK-2
    ....................ucuggauccuuagcucacuuuaag.......   1    0   LT-2
    ....................ucuggauccuuagcucacuuuaag.......   6    0   LT-3
    ....................ucuggauccuuagcucacuuuaag.......   4    0   JCK-2
    ....................ucuggauccuuagcucacuuuaag.......   3    0   JCK-3
    ....................ucuggauccuuagcucacuuuaag.......   5    0   JCK-1
    .....................cuggauccuuagcucacuuua.......   2    0   JCK-3
    .....................cuggauccuuagcucacuuua.......   1    0   JCK-2
    .....................Uuggauccuuagcucacuuuaa.......   4    1   JCK-2
    .....................Uuggauccuuagcucacuuuaa.......   1    1   JCK-1
    .....................Uuggauccuuagcucacuuuaa.......   1    1   JCK-3
    .....................cuggauccuuagcucacuuuaag.......   12   0   JCK-1
    .....................cuggauccuuagcucacuuuaag.......   4    0   JCK-2
    .....................cuggauccuuCgcucacuuuaag.......   1    1   JCK-2
    .....................cuggauccuuagcucacuuuaag.......   16   0   LCK-3
    .....................cuggauccuuagcucacuuuaag.......   10   0   JCK-3
    .....................cuggauccuuagcucacuuuaag.......   16   0   LT-2
    .....................cuggauccuuagcucacuuuaag.......   13   0   LT-1
    .....................cuggauccuuagcucacuuuaag.......   13   0   LT-3
    .....................cuggauccuuagcucacuuuaaga.......   1    0   LT-1
    .....................cuggauccuuagcucacuuuaaga.......   1    0   LT-3
    ......................uggauccuuagcucacuuua.......   2    0   JCK-2
    ......................uggauccuuagcucacuuua.......   1    0   JCK-3
    ......................uggauccuuagcucacuuua.......   2    0   LT-3
    ......................uggauccuuagcucacuuuaag.......   3    0   JCK-3
    ......................uggauccuuagcucacuuuaag.......   5    0   LT-1
    ......................uggauccuuagcucacuuuaag.......   13   0   LT-2
    ......................uggauccuuagcucacuuuaag.......   3    0   JCK-1
    ......................uggauccuuagcucacuuuaag.......   5    0   LT-3
    ......................uggauccuuagcucacuuuaag.......   7    0   JCK-2
```

```
                      Mature                                                                        Star
auuaaacucuuauuuaauuuuucuggauccuuagcucacuuuaagauaucuuccgcccuuuugugggguuauaaagaagugagacuugugaguaaagaguccaaacacguaaguugauuuuggaucagaauaaacaauuuguuuugaccugauuguaagauucuucauucuauuauucugauucauucauaaugcagcgagguggugauccgcucauucuuuguuucguggauuuugcaaauccagccugu
.......................uggauccuuagcucacuuuaag..............................................................................................................................................................................................................   10   0   LCK-3
.......................uggauccuuagcucacuuuaaga.............................................................................................................................................................................................................   1    0   LT-2
.........................gauccuuagcucacuuua................................................................................................................................................................................................................   2    0   JCK-1
.........................gauccuuagcucacuuuaag..............................................................................................................................................................................................................   6    0   JT-3
.........................gauccuuagcucacuuuaag..............................................................................................................................................................................................................   4    0   JCK-1
.........................gauccuuagcucacuuuaag..............................................................................................................................................................................................................   10   0   LT-2
.........................gauccuuagcucacuuuaag..............................................................................................................................................................................................................   4    0   JCK-3
.........................gauccuuagcucacuuuaag..............................................................................................................................................................................................................   2    0   JT-2
.........................gauccuuagcucacuuuaag..............................................................................................................................................................................................................   2    0   LCK-1
.........................gauccuuagcucacuuuaag..............................................................................................................................................................................................................   5    0   JCK-2
.........................gauccuuagcucacuuuaag..............................................................................................................................................................................................................   11   0   LCK-3
.........................gauccuuagcucacuuuaag..............................................................................................................................................................................................................   3    0   LCK-2
.........................gauccuuagcucacuuuaag..............................................................................................................................................................................................................   11   0   LT-3
.........................gauccuuagcucacuuuaag..............................................................................................................................................................................................................   9    0   LT-1
.........................Aauccuuagcucacuuuaag..............................................................................................................................................................................................................   1    1   LT-2
..........................auccuuagcucacuuuaag..............................................................................................................................................................................................................   2    0   LT-3
.......................................................................uaaagaagugagacuugugagu..............................................................................................................................................................   1    0   LT-1
.............................................................................agugagacuugugaguaaagagu.......................................................................................................................................................   2    0   JCK-1
................................................................................gagacuugugaguaaaga.........................................................................................................................................................   1    0   LT-3
.....................................................................................................................................................................cauucuauuauucugauucauu................................................................   1    0   LT-1
.......................................................................................................................................................................................cauucauaaugcagcgagguggugauA.........................................   1    1   LT-1
```
